# Genome Assembly of Alfalfa Cultivar Zhongmu-4 and Identification of SNPs Associated with Agronomic Traits

**DOI:** 10.1016/j.gpb.2022.01.002

**Published:** 2022-01-13

**Authors:** Ruicai Long, Fan Zhang, Zhiwu Zhang, Mingna Li, Lin Chen, Xue Wang, Wenwen Liu, Tiejun Zhang, Long-Xi Yu, Fei He, Xueqian Jiang, Xijiang Yang, Changfu Yang, Zhen Wang, Junmei Kang, Qingchuan Yang

**Affiliations:** 1 Institute of Animal Sciences, Chinese Academy of Agricultural Sciences, Beijing 100193, China; 2 Department of Crop and Soil Sciences, Washington State University, Pullman, WA 99163, USA; 3 School of Grassland Science, Beijing Forestry University, Beijing 100083, China; 4 United States Department of Agriculture-Agricultural Research Service, Plant and Germplasm Introduction and Testing Research, Prosser, WA 99350, USA

**Keywords:** Alfalfa, Autotetraploid, Genome assembly, Resequencing, Genome-wide association study

## Abstract

**Alfalfa** (*Medicago sativa* L.) is the most important legume forage crop worldwide with high nutritional value and yield. For a long time, the breeding of alfalfa was hampered by lacking reliable information on the **autotetraploid** genome and molecular markers linked to important agronomic traits. We herein reported the *de novo* assembly of the allele-aware chromosome-level genome of Zhongmu-4, a cultivar widely cultivated in China, and a comprehensive database of genomic variations based on **resequencing** of 220 germplasms. Approximate 2.74 Gb contigs (N50 of 2.06 Mb), accounting for 88.39% of the estimated genome, were assembled, and 2.56 Gb contigs were anchored to 32 pseudo-chromosomes. A total of 34,922 allelic genes were identified from the allele-aware genome. We observed the expansion of gene families, especially those related to the nitrogen metabolism, and the increase of repetitive elements including transposable elements, which probably resulted in the increase of Zhongmu-4 genome compared with *Medicago truncatula*. Population structure analysis revealed that the accessions from Asia and South America had relatively lower genetic diversity than those from Europe, suggesting that geography may influence alfalfa genetic divergence during local adaption. **Genome-wide association studies** identified 101 single nucleotide polymorphisms (SNPs) associated with 27 agronomic traits. Two candidate genes were predicted to be correlated with fall dormancy and salt response. We believe that the allele-aware chromosome-level genome sequence of Zhongmu-4 combined with the resequencing data of the diverse alfalfa germplasms will facilitate genetic research and genomics-assisted breeding in variety improvement of alfalfa.

## Introduction

Alfalfa (*Medicago sativa* L.), one of the most widely cultivated perennial forage crops worldwide, has been considered as the most valuable forage species because of its high yield and nutritional value [1]. Alfalfa is also relatively tolerant to stress and able to fix nitrogen [2], [3]. The average annual alfalfa hay production in the United States has exceeded 53 million tons in the last three years (Crop Production Summary 2020, United States Department of Agriculture, https://www.ams.usda.gov/market-news/hay-reports). In China, alfalfa hay production in 2019 reached 16.94 million tons from a planting area of approximate 2.32 million ha [4]. With a rapidly increasing demand for milk and ruminant animal production in the developing countries including China, the shortage of high-quality forage crops, particularly alfalfa, has become a limiting factor for the sustainable production of high-quality animals.

Cultivated alfalfa is a cross-pollinated perennial autotetraploid (2n = 4× = 32) species. The complex genetic feature of alfalfa makes it difficult to develop new cultivars using an efficient breeding strategy. Consequently, recurrent phenotypic selection, a time-consuming and labor-intensive method, has been used predominantly for alfalfa breeding. Marker-assisted breeding, an efficient alternative to the traditional phenotypic selection, has been applied and greatly helped in revealing the genetic architecture of the agronomic traits [2], [5]. Several candidate single nucleotide polymorphism (SNP) markers have been identified for quantitative traits, including biomass [6], [7], [8], forage quality [9], [10], [11], [12], and drought/salt tolerance [13], [14], [15]. Quantitative trait loci (QTLs) associated with yield [16], [17], [18] and fall dormancy [19], [20] have also been identified. Additionally, a genomic selection-related study based on the alfalfa biomass yield has been conducted [21]. However, the reference genome used in most of these studies is from *Medicago truncatula*, which is a close relative of alfalfa [22]. Although *M. sativa* and *M. truncatula* are in the same genus, there are some distinct features (*e.g.*, ploidy, overwintering rate, and stress tolerance). Furthermore, some loci of alfalfa are unable to be mapped to the *M. truncatula* genome, reflecting the divergence of alfalfa from its relatives [23], [24]*.* A highly resolved genome is a valuable resource for improving the important agronomic traits of cultivated alfalfa via marker-based breeding.

Assembling chromosome-level genome sequences for autopolyploid or highly heterozygous genomes has historically been a monumental challenge. In 2018, the first allele-aware chromosome-level genome of the autopolyploid *Saccharum spontaneum* was assembled using the ALLHiC algorithm developed by Zhang and his colleagues [25], [26]. Two recently published draft genomes of the autopolyploid alfalfa, Xinjiangdaye and Zhongmu-1, provide valuable information for related studies of alfalfa [27], [28]. The Xinjiangdaye genome was assembled at the allele-aware chromosome level using the ALLHiC algorithm, whereas the Zhongmu-1 genome was merely assembled at the haploid chromosome level. Compared with several other plants such as maize and rice, the genomic information of cultivated alfalfa is still limited. In this study, we assembled the allele-aware chromosome-level genome sequence of the Chinese alfalfa cultivar Zhongmu-4, which is one of the most common alfalfa cultivars grown in northern China because of its high yield and salt tolerance. Zhongmu-4 alfalfa was bred by us and released in 2011. Advantages of this cultivar include its greater root system, large leaf area, high nutritional value (about 20% crude protein content), fast regeneration, high yield, and salt tolerance. Zhongmu-4 alfalfa is widely cultivated in the Huang-Huai-Hai region of China. The mean annual hay yield can reach 15.0–17.4 t/ha, which is about 10% greater than that of Zhongmu-1. By the end of 2020, the total planting area of Zhongmu-4 has exceeded 200,000 ha. Moreover, in the present study, we evaluated 93 agronomic traits of 220 alfalfa accessions from a global germplasm resource. On the basis of our assembled genome sequence and genotyping by whole-genome sequencing, 111,075 SNP markers were screened. Several SNPs associated with agronomic traits (*e.g.*, salt tolerance and fall dormancy) were identified by genome-wide association studies (GWAS).

## Results

### Allele-aware genome assembly and annotation

**Supplementary Figure S1:**
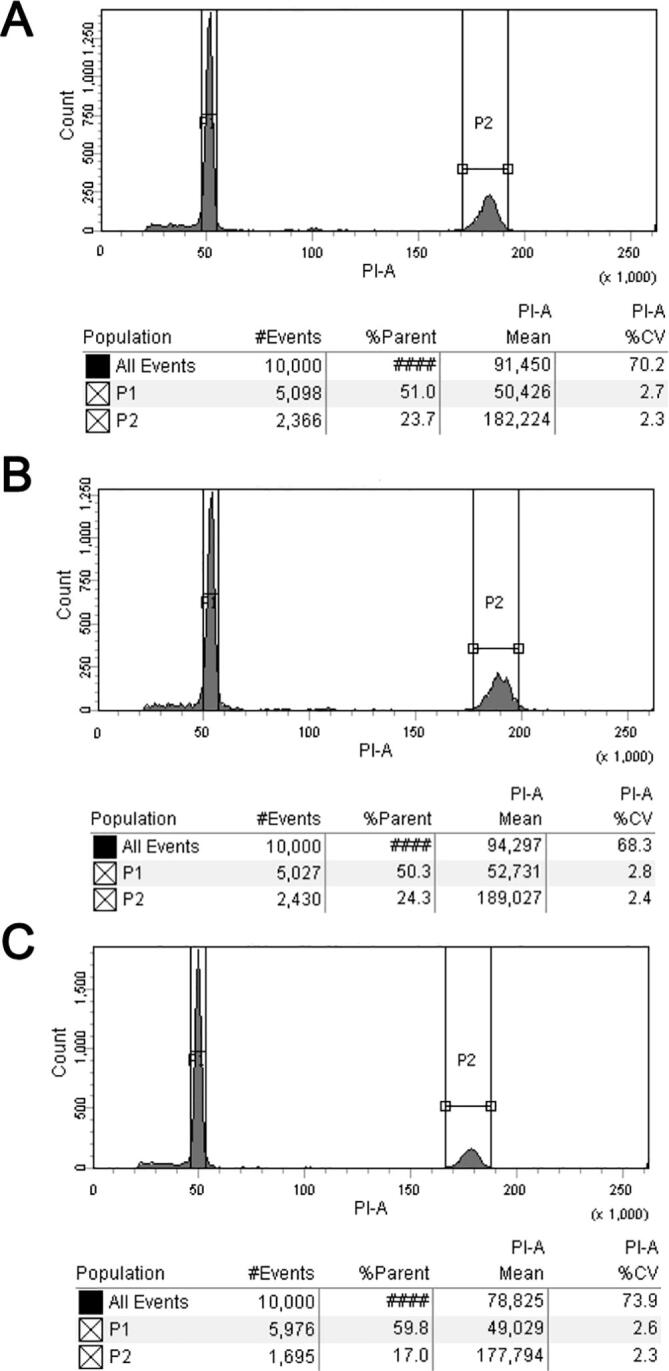
**PI-A values of *M. truncatula* ecotype Jemalong A17 and *M. sativa* cultivar Zhongmu-4 determined by flow cytometry A**. The result of the first independent repeat. **B**. The result of the second independent repeat. **C**. The result of the third independent repeat. P1 and P2 represent the PI-A values of Jemalong A17 and Zhongmu-4. The average of PI-A value ratios (Zhongmu-4/Jemalong A17) is about 3.61. PI-A, propidium iodide adsorbing; CV, coefficient of variance.

**Supplementary Figure S2:**
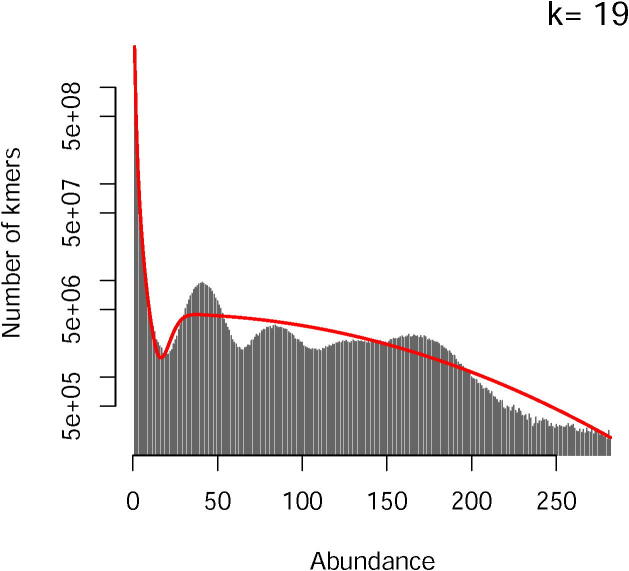
***K*-mer frequency analysis result** The haploid genome size of Zhongmu-4 was estimated as 1,480,963,752 bp by KmerGenie (*K* = 19), and thus the estimated whole genome size was about 2962 Mb (1,480,963,752 bp × 2).

Flow cytometry analysis revealed that the DNA peak ratio of Zhongmu-4 to *M. truncatula* ecotype Jemalong A17, a close relative of alfalfa with an estimated haploid genome size of 425 Mb (1C) [22], was about 3.61 (Figure S1), indicating that the tetraploid genome of Zhongmu-4 was approximately 3068 Mb (2C) (Table S1). The estimation was close to the genome size (3371 Mb) obtained based on the 2C value (3.44 pg) in the Plant DNA C-values database. Using the 168 Gb Illumina 150 bp paired-end genome sequencing reads, the Zhongmu-4 genome was surveyed and the tetraploid genome size was estimated as 2962 Mb by KmerGenie according to the *K*-mer frequency (*K* = 19) (Figure S2). Based on the aforementioned estimates, the estimated Zhongmu-4 genome size was set at 3.1 Gb for further analyses.

**Figure 1:**
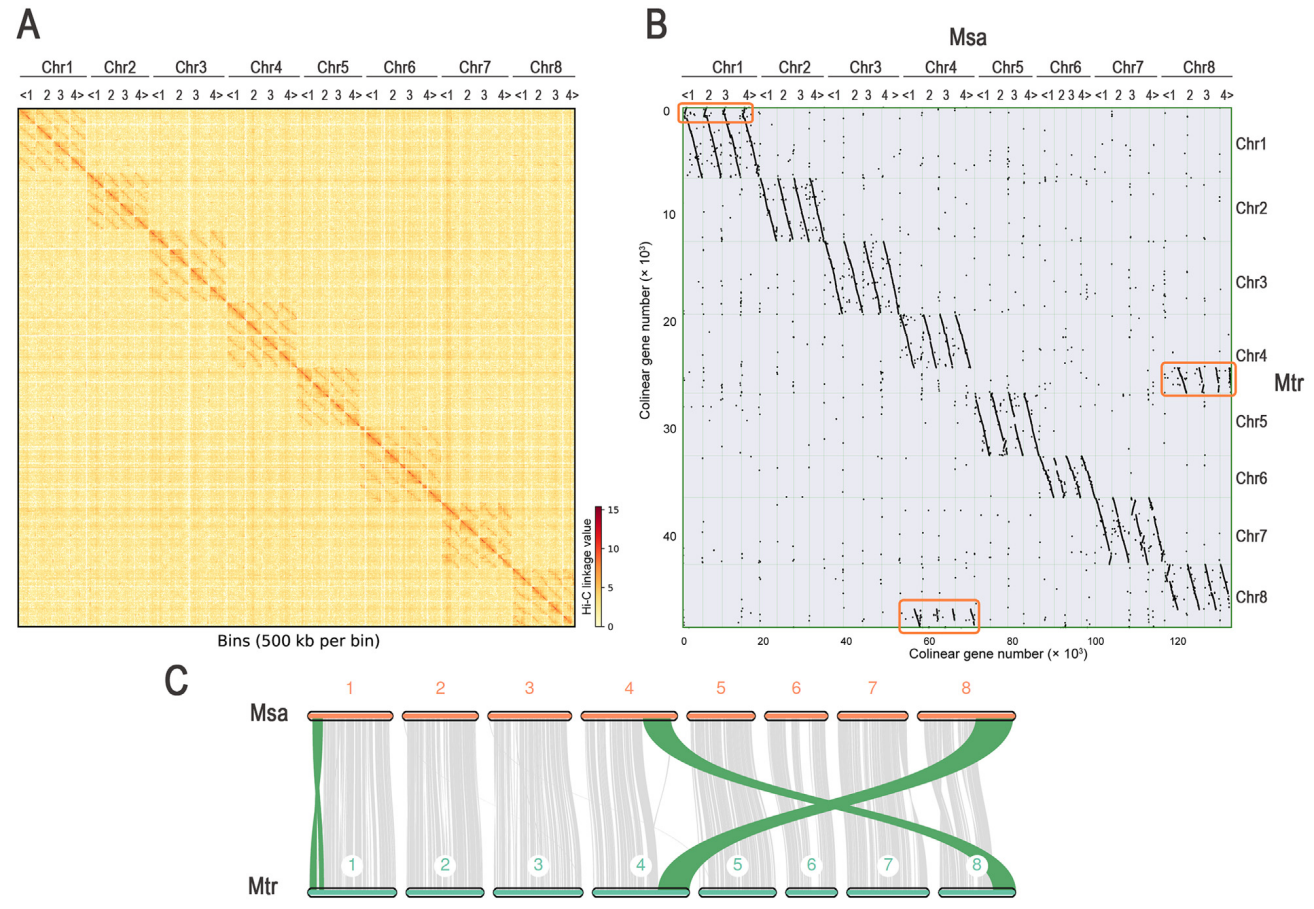
**Hi-C linkage and synteny analyses** **A.** Hi-C linkage plot of 32 chromosomes in the Zhongmu-4 genome. **B.** Alignment and synteny analysis of four subgenomes of *Medicago sativa* cultivar Zhongmu-4 and the genome of the *M. truncatula* ecotype Jemalong A17. The translocation between Chr4 and Chr8 and the inversion in Chr1 were labeled by orange boxes. **C.** Synteny linkage plot of the first set of monoploid chromosomes of Zhongmu-4 and Jemalong A17. Hi-C, high-through chromosome conformation capture; Chr, chromosome; Msa, *M. sativa* cultivar Zhongmu-4; Mtr, *M. truncatula* ecotype Jemalong A17.

**Supplementary Figure S3:**
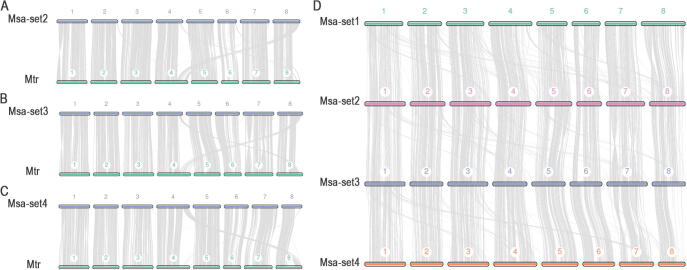
**Synteny analysis of Zhongmu-4 subgenomes and Jemalong A17 genome A**. Plot of the synteny between the set 2 subgenome of Zhongmu-4 and the genome of Jemalong A17. **B**. Plot of the synteny between the set 3 subgenome of Zhongmu-4 and the genome of Jemalong A17. **C**. Plot of the synteny between the set 4 subgenome of Zhongmu-4 and the genome of Jemalong A17. **D**. Plots of the synteny within the set 1, set 2, set 3, and set 4 subgeome.

The 262 Gb continuous long reads (CLRs) generated by the PacBio Sequel sequencing system (Table S2) were used for the self-correction and assembly by Canu. Approximately 2.74 Gb contigs, accounting for 88.39% of the estimated genome size, were assembled with contig N50 of 2.06 Mb and GC content of 34.2% (Table S3). The assembled contig sequences were corrected and polished using 168 Gb Illumina paired-end sequences, and then preliminarily assembled using 3D-DNA, yielding 49,967 corrected contigs (Table S3). The corrected and polished contigs were imported to the polyploid genome assembly program ALLHiC to build an allele-aware chromosome-scale genome using high-through chromosome conformation capture (Hi-C) paired-end reads. The final assembled genome included 2.56 Gb contigs anchored to 32 pseudo-chromosomes (super-scaffolds), which represented eight groups of homologous chromosomes with four sets of monoploid chromosomes, and 0.18 Gb unanchored contigs. The assembly quality was assessed according to the synteny analysis, Hi-C contact matrix, benchmarking universal single-copy orthologs (BUSCO), and the long terminal repeat (LTR) assembly index (LAI). The Hi-C linkage plot indicated that the chromosome groups were distinct (Figure 1A; Table S4). The sequence alignment and synteny analysis revealed high collinearity between the individual Zhongmu-4 subgenome and the Jemalong A17 genome (Figure 1B and C, Figure S3). Approximately 98.4% of BUSCOs (1588 out of 1614) were detected in the assembled genome (Table S5). The LAI of the assembled genome was about 13.85, indicating that Zhongmu-4 genome assembly reached the Reference level of Ou’s classification system [29]. Thus, these results indicate the high quality of the assembled Zhongmu-4 genome.

**Figure 2:**
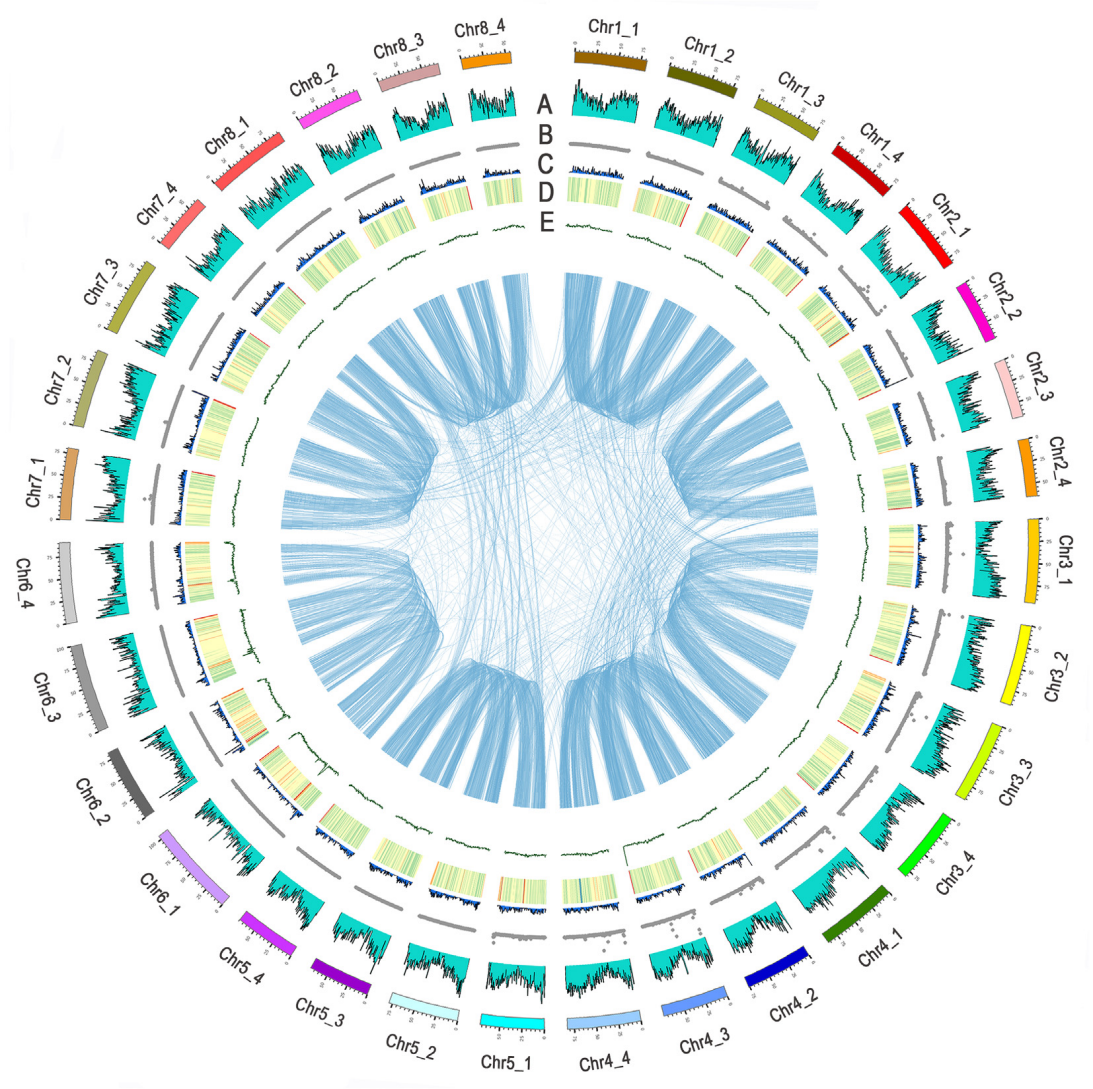
**Distribution of Zhongmu-4 genomic features** **A.** Density of annotated genes. **B.** Density of filtered SNPs in the GWAS population. **C.** Density of syntenic paralogous gene pairs with a Ka/Ks ratio > 1. **D.** Density of repetitive sequences. **E.** GC content density. Synteny blocks are linked in the center. The outermost bars represent the length (Mb) of chromosomes. SNP, single nucleotide polymorphism; GWAS, genome-wide association study; Ka, the number of nonsynonymous substitutions per nonsynonymous site; Ks, the number of synonymous substitutions per synonymous site.

A total of 146,704 protein-coding genes were identified in the assembled allele-aware genome, and 136,467 genes (93.02%) were functionally annotated according to the InterPro, eggNOG, and Kyoto Encyclopedia of Genes and Genomes (KEGG) databases. The gene density in the 32 monoploid chromosomes is presented in Figure 2 and Table S6. Using the first set of subgenomes as the reference, 34,922 allelic genes were identified in the four sets of allele-aware chromosomes (10,704 four-allele genes, 9526 three-allele genes, 7129 two-allele genes, and 7563 one-allele genes).

### Repetitive elements drive genome expansion of alfalfa

**Supplementary Figure S4:**
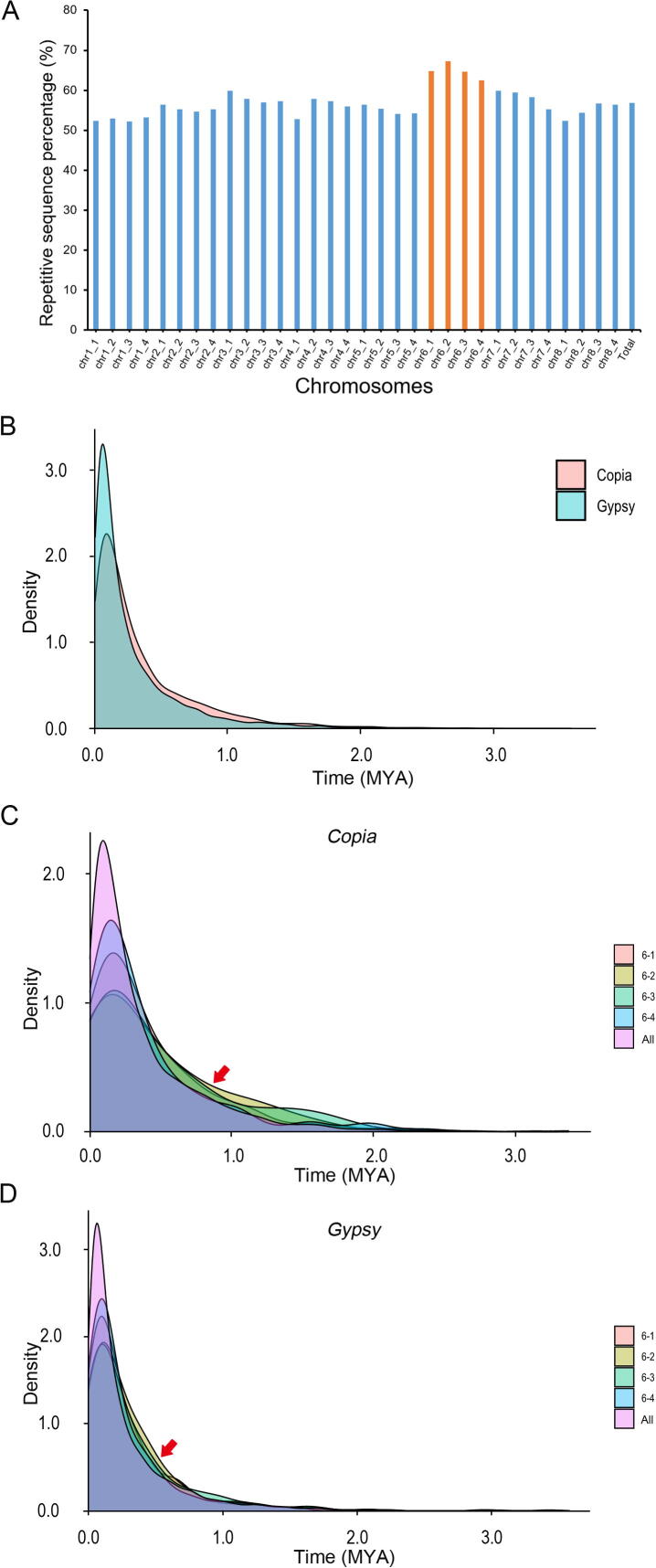
**Repetitive element analysis A**. Repetitive sequences proportions in each chromosome and the whole genome of Zhongmu-4. **B**. Density of insert time of Copia and Gypsy LTR transposable elements of Zhongmu-4. **C**. Density of insert time of Copia LTR transposable elements in the whole genome and group 6 chromosomes. **D**. Density of insert time of Gypsy LTR transposable elements in the whole genome and group 6 chromosomes. LTR, long terminal repeats.

A total of 1558 Mb repetitive sequences were identified, accounting for 56.80% of the assembled Zhongmu-4 genome (Figure 2), of which 49.28% were LTR transposable elements (TEs), including Ty1-*Copia* superfamily retrotransposons (10.93%), Ty3-*Gypsy* superfamily retrotransposons (18.33%), and unclassified LTR elements (20.02%). The remaining repetitive sequences were mainly long or short interspersed nuclear element segments (LINEs or SINEs), DNA elements, and simple repeats. In contrast, *M. truncatula* contained 181 Mb repetitive sequences (43.94% of the genome), including 76 Mb LTR TEs, accounting for 18.36% of the genome. Thus, the proportion of repetitive sequences, especially LTR elements, was larger in Zhongmu-4 than in *M. truncatula*. These results suggest that the accumulation of LTR is probably one of the main reasons for the expansion of alfalfa genome. Regarding individual chromosomes, the group 6 chromosomes (Chr6_1 to Chr6_4) had a larger proportion of repetitive sequences than the other chromosomes (Figures 2, Figure S4A). These observations may explain, at least to some extent, why the assembly quality of chromosome 6 was relatively low with fewer protein-coding genes detected compared with the other chromosomes (Table S6). The distribution of LTR TE insertion time indicated that an LTR TE burst occurred in the Zhongmu-4 genome within the last one million years (Figure S4B). The LTRs in the group 6 chromosomes were more active than those in the whole genome (Figure S4C and D).

### Gene family evolution and whole-genome duplication

**Figure 3:**
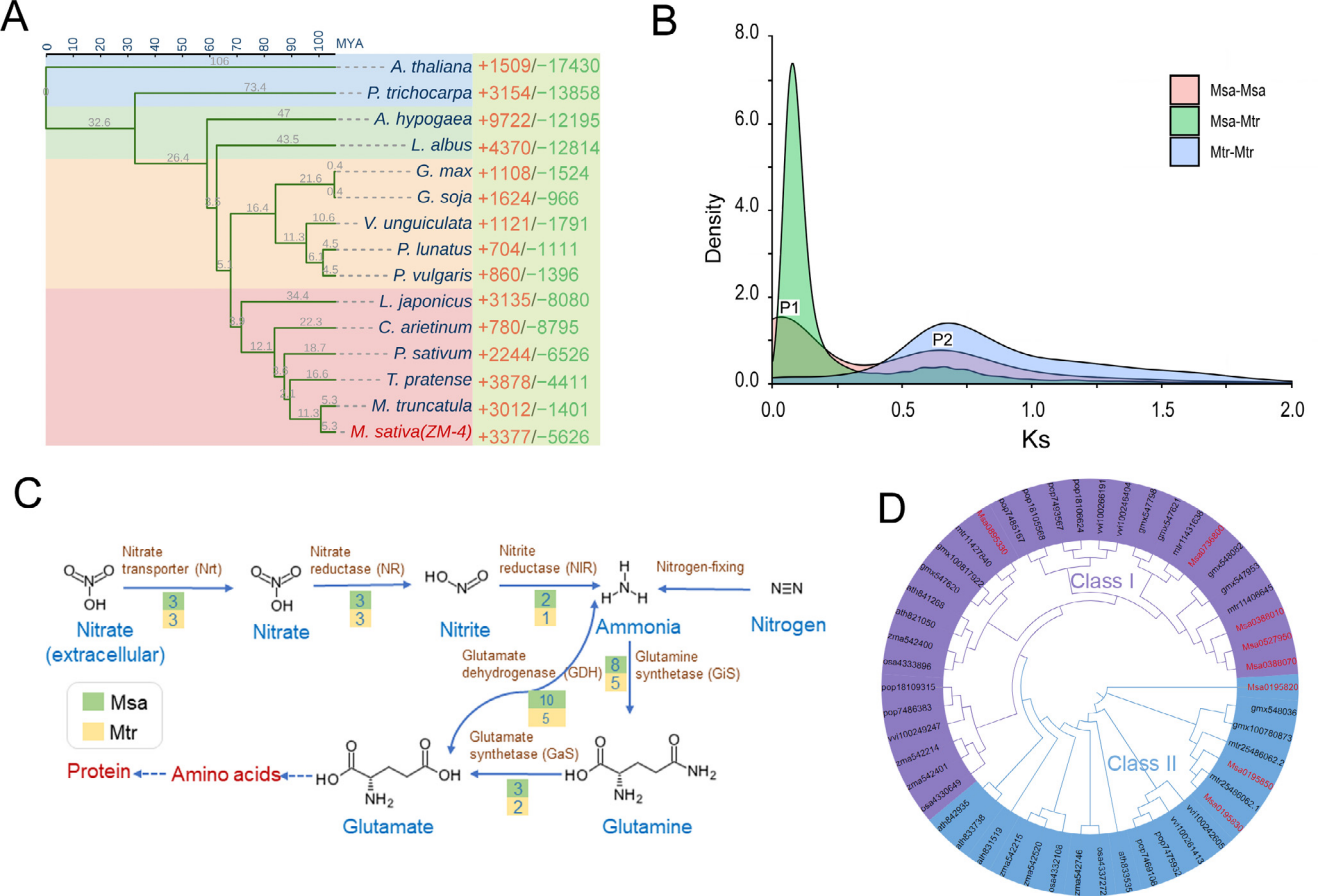
**Comparative genome analysis and identification of nitrogen metabolism-related genes** **A.** Phylogenetic tree of Zhongmu-4 and 14 dicotyledonous species. “+” and “−” indicate expanded (red) and contracted (green) gene families, respectively. **B.** Density distribution of Ks values of the syntenic paralogous genes in/between the first subgenome of Zhongmu-4 and the genome of Jemalong A17. **C.** Key enzymes in the nitrogen metabolism pathway. The number in each colored block indicated the number of enzyme-coding genes. **D.** Phylogenetic tree of glutamine synthetases from Zhongmu-4 (Msa), Jemalong A17 (mtr), *G. max* (gmx), *A. thaliana* (ath), *Z. mays* (zma), *O. sativa* (osa), *V. vinifera* (vvi), and *P. trichocarpa* (pop). The eight glutamine synthetases from Zhongmu-4 were marked in red. The NCBI-Gene IDs were used to represent the glutamine synthetases from other seven species. MYA, million years ago.

**Supplementary Figure S5:**
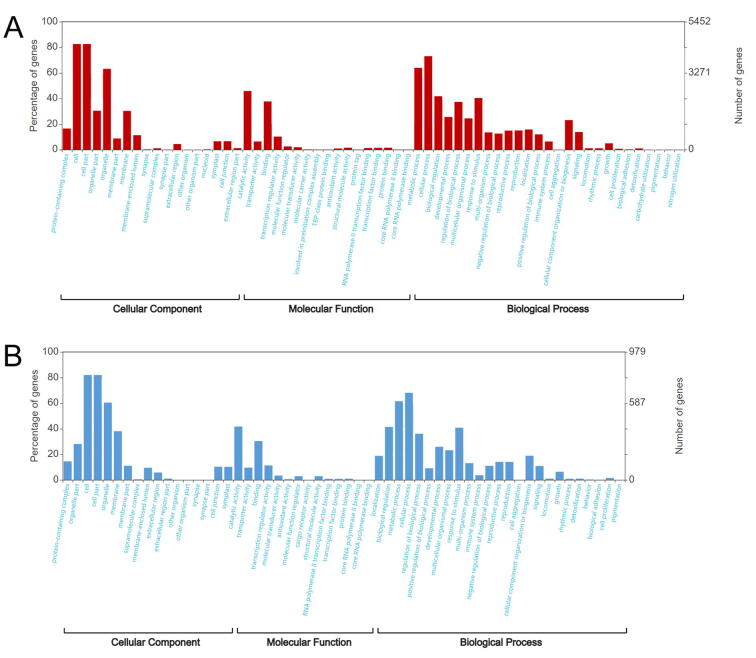
**GO annotation analysis of expanded and contracted genes A**. GO annotation of expanded genes in Zhongmu-4. **B**. GO annotation of contracted genes in Zhongmu-4.

A phylogenetic tree was constructed using the 28 single-copy orthologous genes identified in *M. sativa* (the first subgenome of Zhongmu-4 was used), *Arabidopsis thaliana*, *Populus trichocarpa,* and 12 legume species (Figure 3A). The time of the divergence between *A. thaliana* and *M. sativa*, 106 million years ago (MYA), was used as a reference. *M. sativa* was more closely related to the legume species than to *A. thaliana* or *P. trichocarpa* (Figure 3A). Moreover, it diverged from *M. truncatula* approximately 5.3 MYA. The gene family size changed in the 15 selected species during evolution, with 3377 expanded gene families and 5626 contracted gene families in the first subgenome of Zhongmu-4 (Figure 3A). Gene Ontology (GO) annotation results showed that both the expanded and contracted genes were involved in 25 common biological process terms, predominantly in metabolic process and cellular process. Interestingly, some expanded genes were annotated in two biological process terms, nitrogen utilization and carbohydrate utilization (Figure S5). According to the gene numbers of the expanded and contracted families, PIF1-like helicase, TIR-NBS-LRR disease resistance protein, and heat shock 70 protein are the most notable expanded gene families, while MADS-box transcription factor, transcription termination factor, and F-box protein interaction domain protein are the most notable contracted gene families.

Synteny block analysis is often conducted to assess the assembly quality and investigate the evolution of related species. Following the protein sequence alignment, the syntenic blocks revealed that the four Zhongmu-4 subgenomes had strong collinear relationships with the Jemalong A17 genome. A translocation between Chr4 and Chr8 and a consistent inversion in Chr1 were detected (Figure 1B and C, Figure S3). The detection of the translocation and inversion was consistent with the previous study [27].

**Supplementary Figure S6:**
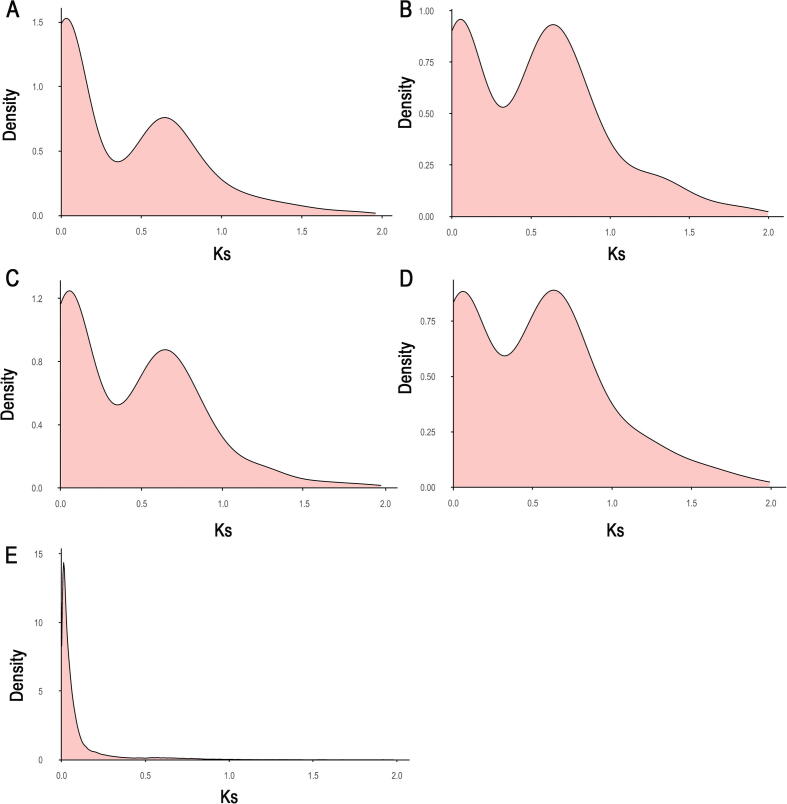
**Density of Ks value for the syntenic paralogous genes in each subgenome and allele-aware genome of Zhongmu-4 A**. Ks value density of set 1 subgenome of Zhongmu-4**. B**. Ks value density of set 2 subgenome of Zhongmu-4**. C**. Ks value density of set 3 subgenome of Zhongmu-4. **D**. Ks value density of set 4 subgenome of Zhongmu-4**. E**. Ks value density of allele-aware genome of Zhongmu-4.

We analyzed the number of synonymous substitutions per synonymous site (Ks), the number of nonsynonymous substitutions per nonsynonymous site (Ka), and the Ka/Ks ratio in Zhongmu-4. The Ks value densities of Zhongmu-4 and Jemalong A17 revealed one overlapping peak P2 (Msa-Msa: 0.65), implying that they experienced a common ancient whole-genome duplication event before their divergence (Figure 3B, Figure S6). The Ks peak P1 (Msa-Msa: 0.04) of Zhongmu-4 was mainly caused by tandem repeat, segmental duplication, and high-frequency recombination of some allelic chromosome regions. The distribution of genes with Ka/Ks > 1 (between syntenic paralogous gene pairs) is presented in Figure 2. There was no significant difference among the allelic chromosomes.

### Nitrogen metabolism-related gene families expand in Zhongmu-4

A BLASTP search identified genes encoding six key enzymes [*e.g.*, glutamine synthetase (GiS)] in the nitrogen metabolism pathway in the first subgenome of Zhongmu-4. The genes encoding these enzymes identified in Zhongmu-4 and seven other species are listed in Table S7. The number of genes encoding nitrite reductase (NIR), GiS, glutamate dehydrogenase (GDH), or glutamate synthetase (GaS) was significantly larger in Zhongmu-4 than in Jemalong A17 (Figure 3C), implying that these gene families expanded after the divergence from Jemalong A17. The genes encoding GDH or GiS were more abundant in Zhongmu-4 than in six other analyzed species (Table S7). A phylogenetic tree constructed based on the protein sequence of GiS from Zhongmu-4 and seven other species revealed that the GiS proteins were obviously divided into two clusters (Figure 3D).

### SNP identification and population structure differentiation

**Figure 4:**
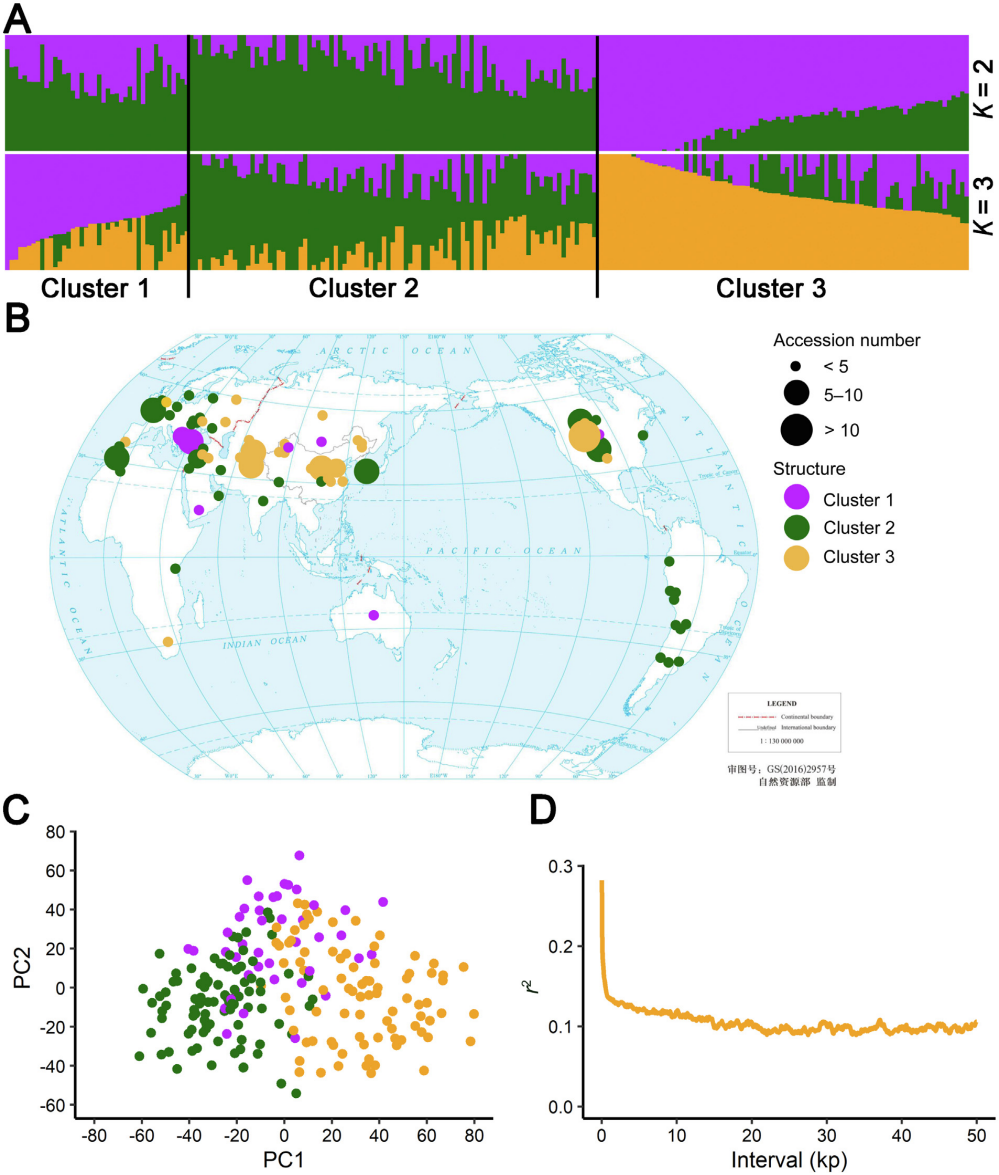
**Analyses of the population structure and LD** **A.** Population structure cluster plot. **B.** Global distribution of the 220 accessions included in the population cluster plot. The base world map is from China Standard Map Service (http://bzdt.ch.mnr.gov.cn/). **C.** PCA involving PC1 and PC2 pairwise scatter plots. **D.** LD decay within 50 kb. PCA, principal component analysis; PC, principal component; LD, linkage disequilibrium.

**Supplementary Figure S7:**
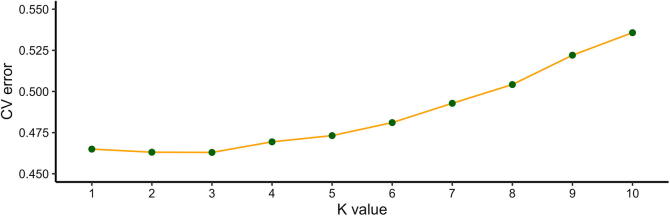
**Cross-validation error plot (K = 1 to K = 10)** The best K value is 3 (lowest CV error). CV, cross-validation.

To explore SNPs related to alfalfa agronomic traits, 220 accessions collected from six continents were genotyped by whole-genome sequencing. About 29.6 million SNPs detected using BWA-SAMtools-VarScan pipeline were filtered according to the following criteria: missing value > 10%, minimum mean read depth > 5, and minor allele frequency (MAF) > 0.05. On the basis of 111,075 remaining SNP markers, the subgroup population structure was estimated using Admixture, with a *K* value of 2–10. The 220 accessions were divided into three clusters according to the cross-validation (CV) error (Figure 4A, Figure S7). Cluster 1 primarily comprised accessions from Europe, Cluster 2 included accessions with diverse backgrounds, and Cluster 3 mainly consisted of accessions from Asia (Figure 4B). A principal component analysis (PCA) showed similar results (Figure 4C), and the variations explained by the top three principal components (PCs) were 3.5%, 1.8%, and 1.5%, respectively, reflecting the relatively weak population structure of the alfalfa accessions. Linkage disequilibrium (LD) analysis indicated that a decay was observed at 15 kb in the whole genome with *r*^2^ < 0.1 (Figure 4D).

### GWAS for 93 phenotypic traits

**Supplementary Figure S8:**
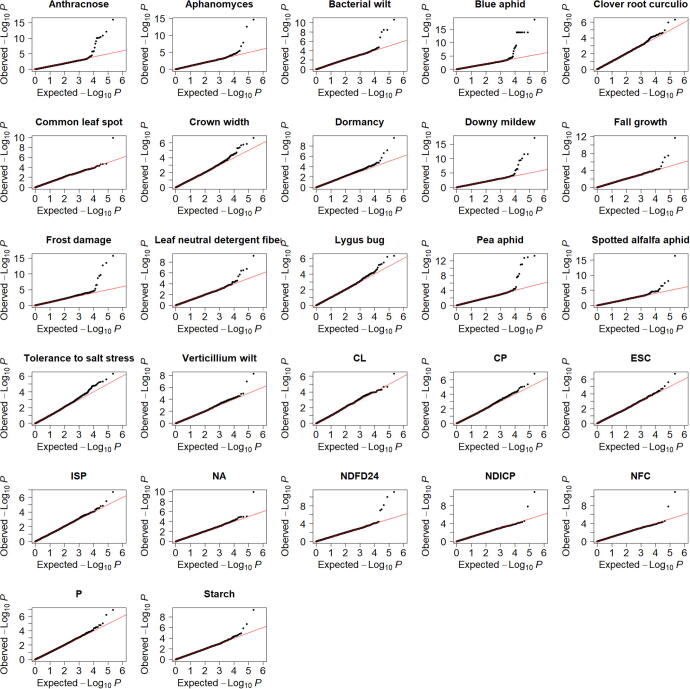
**QQ plots of 27 agronomic traits with significantly associated SNPs** The trait names are provided at the top of the QQ plots. CL, chlorine; CP, crude protein; ESC, ethanol soluble carbohydrates; ISP, insoluble protein; NA, sodium; NDFD24, neutral detergent fiber digestibility in 24 hours; NDICP, neutral detergent insoluble crude protein; NFC, nonfiber carbohydrates; P, phosphorus.

To identify candidate loci/genes related to important alfalfa agronomic traits, 93 traits (35 collected in this study and 58 collected from the USDA GRIN database) were analyzed by a GWAS using the mapping panel for the 220 accessions. A total of 101 SNPs were identified to be associated with 27 traits, including quality-related traits (*e.g.*, crude protein content and neutral detergent insoluble crude protein content), disease-related traits (*e.g.*, resistance to aphanomyces root rot and verticillium wilt), and growth-related traits (*e.g.*, dormancy height and fall growth). Our results showed that the average number of SNPs associated with a trait was 4. Notably, the resistance to blue alfalfa aphid was associated with the most SNPs (*n* = 14), while nine traits were associated with only a single SNP (Table S8; Figure S8). Trait-associated markers are valuable resources for the marker-assisted selection during alfalfa breeding.

**Figure 5:**
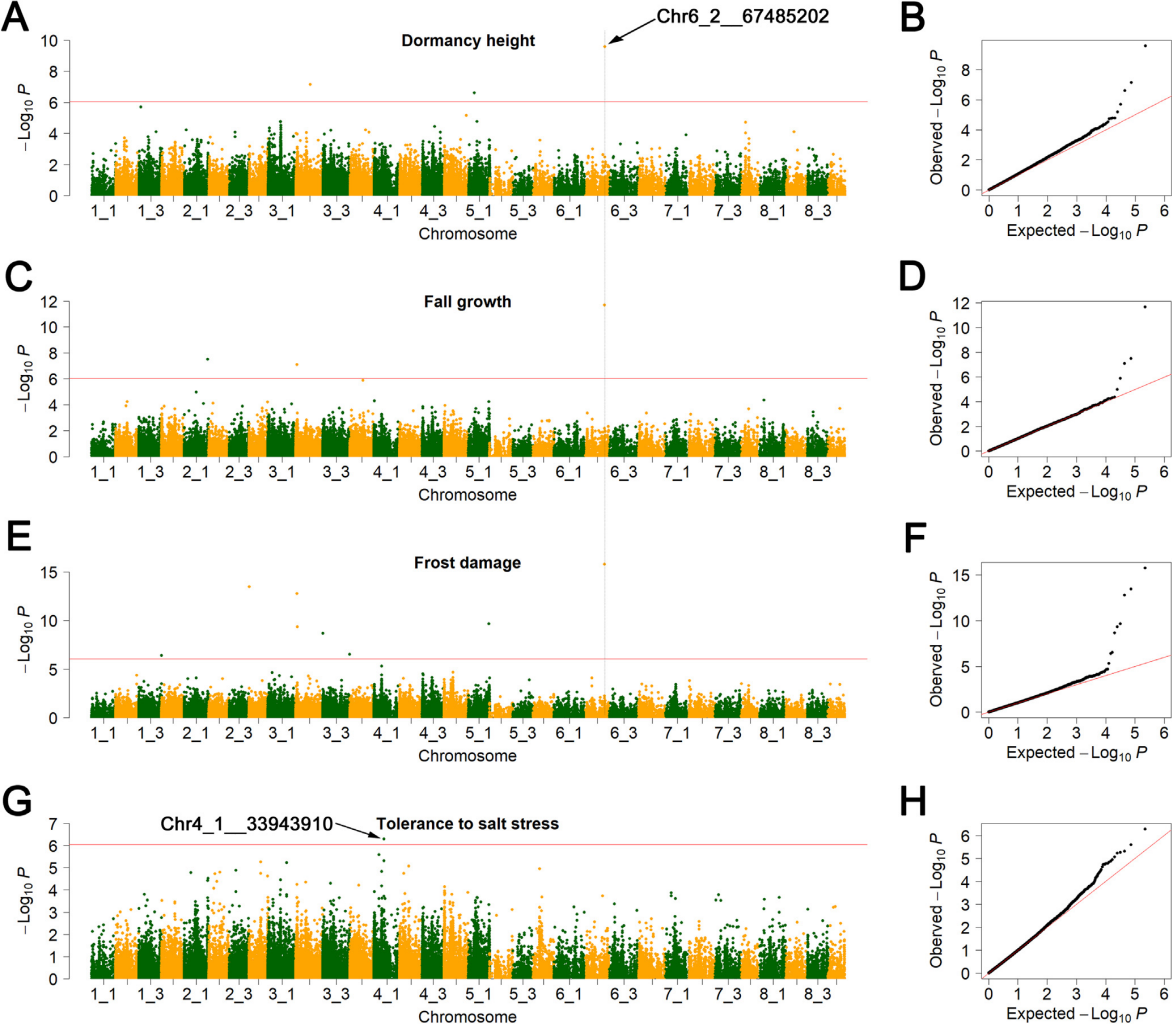
**Manhattan plot and QQ plot of the SNPs associated with fall dormancy and salt tolerance** **A.** Manhattan plot of the SNPs associated with dormancy height. **B.** QQ plot of the SNPs associated with dormancy height. **C.** Manhattan plot of the SNPs associated with fall growth. **D.** QQ plot of the SNPs associated with fall growth. **E.** Manhattan plot of the SNPs associated with frost damage. **F.** QQ plot of the SNPs associated with frost damage. In (A), (C), and (E), the dashed line indicates the location of a SNP associated with all the three traits, whereas the arrow indicates its locus. **G.** Manhattan plot of the SNPs associated with salt stress tolerance. **H**. QQ plot of the SNPs associated with salt stress tolerance. The GWAS was performed using the Blink function of the GAPIT3 software. The significance threshold was set as *P* = 9.00 × 10^−7^.

**Figure 6:**
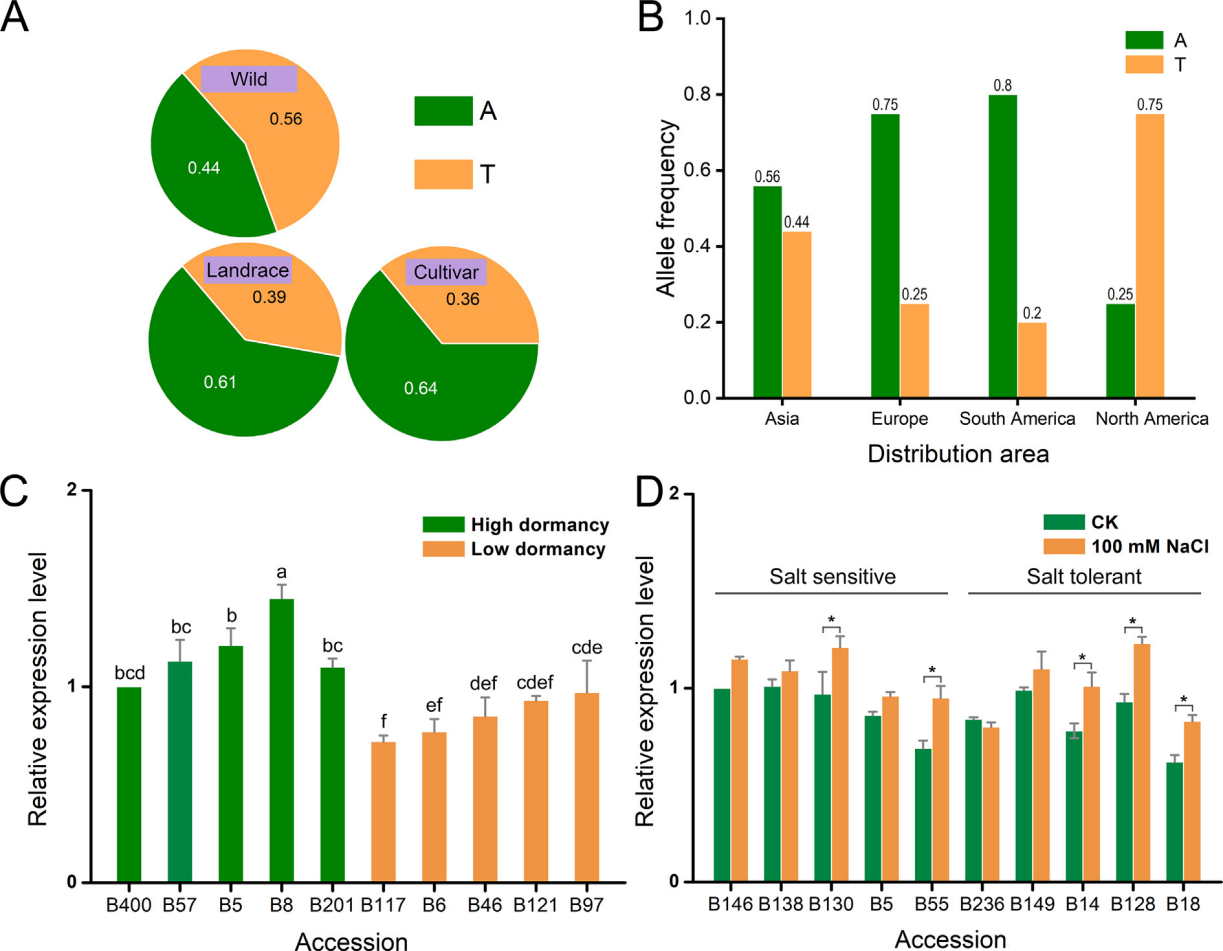
**The allele frequency of a multi-effect SNP and the relative expression of two candidate genes in representative accessions** **A.** Allele frequency of a SNP (Chr6_2__67485202) in wild materials, landraces, and cultivars of alfalfa. **B.** Allele frequency of Chr6_2__67485202 in cultivars from Asia, Europe, South America, and North America. **C.** Relative expression analysis (qRT-PCR) of a candidate gene (Msa0924940) encoding a NADH dehydrogenase for SNP (Chr6_2__67485202) in representative accessions. Data were arbitrarily normalized against the data of accession B400. The different letters refer to the significant differences at *P* < 0.05 (Duncan’s multiple range test) **D.** Relative expression analysis (qRT-PCR) of MYM-type zinc finger protein gene (Msa0544870) in representative salt-tolerant and salt-sensitive accessions. Data were arbitrarily normalized against the control data of accession B146. *, *P* < 0.05 (Student’s *t*-test). CK, control data; NADH, nicotinamide adenine dinucleotide; MYM, myeloproliferative and mental retardation.

**Supplementary Figure S9:**
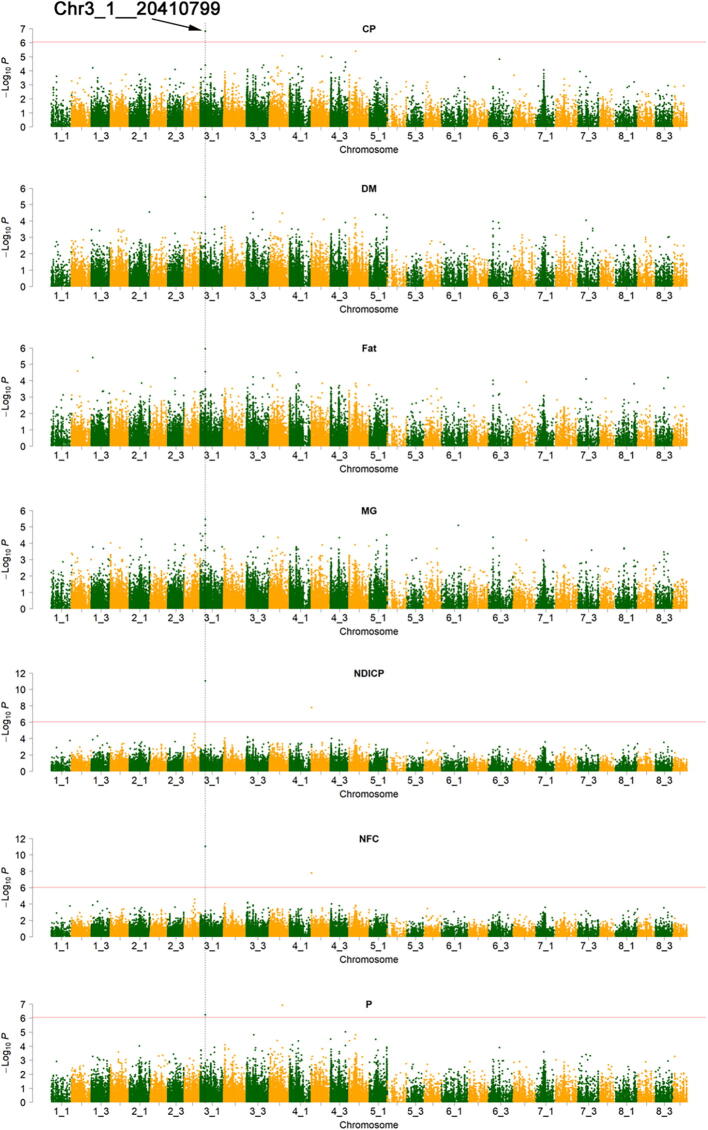
**Manhattan plots of seven nutrition-related traits** The chromosome name is provided on the X-axis. The log_10_(*P*) value is provided on the Y-axis. The black dash line represents the significant *P* value. DM, digestible energy; MG, magnesium.

We also observed several SNPs individually associated with multiple traits. For example, the SNP at 20.41 Mb on chromosome 3_1 (Chr3_1__20410799) was associated with the crude protein content, neutral detergent insoluble crude protein content, non-fiber carbohydrate content, and phosphorus content (Table S8; Figure S9), which are key indexes for hay quality and nutritional value of alfalfa. We also identified a locus at 67.48 Mb on chromosome 6_2 (Chr6_2__67485202) associated with dormancy height, fall growth, and frost damage (Figure 5A–F), which are closely related to one another. An allele frequency analysis revealed that the A allele of Chr6_2__67485202, which was significantly associated with high dormancy, was present more frequently in the selected cultivated alfalfa (0.64) and landraces (0.61) relative to the wild materials (0.44) (Figure 6A). Geographically, the A allele frequency was higher in the cultivars from Asia (0.56), Europe (0.75), or South America (0.8) than those from North America (0.25) (Figure 6B). Seven protein-coding genes were annotated within 15 kb (LD = 15 kb, *r*^2^ = 0.1) upstream and downstream of the SNP in the Zhongmu-4. The expression data demonstrated that one candidate gene [Msa0924940, nicotinamide adenine dinucleotide (NADH) dehydrogenase] had higher expression in high-dormancy accessions than those with low-dormancy (Figure 6C). Regarding the SNP (at 33.94 Mb on chromosome 4_1) significantly associated with salt stress tolerance (Figure 5G and H), the expression of one of the two candidate genes [Msa0544870, myeloproliferative and mental retardation (MYM)-type zinc finger protein gene; Msa0544860, TPD1 protein homolog 1-like protein gene] was up-regulated by salinity treatment (100 mM NaCl), and the salt-tolerant accessions showed higher induction of Msa0544870 compared to the salt-sensitive ones (Figure 6D).

## Discussion

**Supplementary Figure S10:**
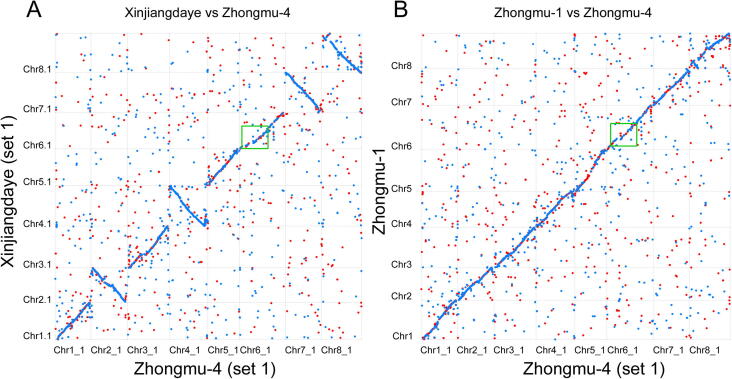
**Whole genome alignment analysis of Zhongmu-4, Xinjiangdaye, and Zhongmu-1 A**. Alignment of Zhongmu-4 and the set 1 subgenome of Zhongmu-1**. B**. Alignment of Xinjiangdaye and the set 1 subgenome of Zhongmu-1.

In this study, we assembled the allele-aware chromosome-scale genome of an autotetraploid Chinese alfalfa cultivar Zhongmu-4 by using the third-generation sequencing and Hi-C sequencing technologies as well as allele-aware chromosome-level assembly algorithm. This is the second allele-aware chromosome-level reference genome assembled for an autotetraploid alfalfa species; the first was constructed for the Xinjiangdaye alfalfa landrace [28]. Shen et al. recently assembled the chromosome-level genome sequence of alfalfa cultivar Zhongmu-1, but only to the haploid chromosome level [27]. The assembly quality parameters N50, BUSCO, and anchor rate for the Zhongmu-4 assembly were better than those for the earlier allele-aware Xinjiangdaye genome assembly. Additionally, the Zhongmu-4 genome was shorter than the Xinjiangdaye genome (Table S9). These differences may be related to the diversity in the sequencing strategies used for analyzing these two species. More specifically, the PacBio CLR mode was used for the Zhongmu-4 genome sequencing, whereas the Nanopore and PacBio circular consensus sequencing (CCS) mode were used for the Xinjiangdaye genome sequencing. Nucleic acid sequence alignment of Zhongmu-4 subgenomes, Zhongmu-1 genome, and Xinjiangdaye subgenomes revealed that they had high similarity on the whole genome (Figure S10). In some regions of chromosomes, especially in chromosome 6, there were some alignment gaps, which may be caused by the high abundance of TEs in this chromosome. Because of outcrossing and self-incompatibility, alfalfa is a highly heterozygous species, with genotypic variations between alfalfa cultivars and individual plants. Accordingly, we assembled the allele-aware chromosome-level genome of Zhongmu-4, which is a representative cultivar of the Zhongmu series. Compared with the previously sequenced haploid genome of Zhongmu-1, our allele-aware genome of Zhongmu-4 has more BUSCOs and provides more information regarding sequence variations and alleles. The rapid development of sequencing technologies has enabled the construction of multiple high-quality reference genomes for some important crops, including maize and rice. These reference genomes have been used for the comprehensive identification of genome-wide variations and for functional genomics investigations and breeding practices. However, the genome resources available for alfalfa are still relatively limited. In this study, we generated a new high-quality reference genome resource for future research in alfalfa.

Alfalfa is an important legume forage crop cultivated worldwide for the subsequent use in livestock production because of its high protein content and high nutritional value [1]. Its symbiosis with *Rhizobium* bacterial species and ability to fix nitrogen are critical for protein synthesis [3]. Nitrogen is an important nutrient for plant growth and development, but it is also a crucial component of biological macromolecules, *e.g.*, nucleic acids and proteins [30]. Plants obtain nitrogen from the soil mainly via the uptake of nitrogenous compounds, *e.g.*, NO_3_^−^ and NH_4_^+^, through their roots. Inorganic nitrogen must be processed into glutamine by the nitrogen metabolism pathway for further transformation and use (Figure 3C). GiS and GDH are key enzymes responsible for converting inorganic nitrogen into an organic form [31]. Interestingly, our analyses revealed that there are more GiS and GDH genes in the Zhongmu-4 genome than in the genomes of other plant species, including corn, rice, and *M. truncatula*; however, the soybean genome contains a similar number of these genes (Table S7). This is consistent with the ability of Zhongmu-4 plants to absorb nitrogen and efficiently synthesize proteins.

The lack of a reference genome was a limiting factor for earlier molecular research on alfalfa, *e.g.*, GWAS, QTL analyses, and investigations regarding evolution. Traditional methods used for breeding new alfalfa cultivars with improved traits are time-consuming processes and require the screening of large populations. A molecular marker-assisted breeding approach involving a high-quality reference genome can shorten the breeding cycle and increase breeding efficiency. To evaluate the genome quality and identify agronomic traits associated with molecular markers, we used the assembled genome of Zhongmu-4 as the reference genome for a GWAS and an analysis of the population structure of 220 accessions.

Alfalfa genetic improvements to date have mainly relied on recurrent phenotypic selection and outcrossing. The genetic exchange among individual alfalfa plants is generally unavoidable in alfalfa-growing regions [2]. Distinguishing accessions according to geographical origins is more difficult for alfalfa than for self-pollinated plants (*e.g.*, soybean and rice) [32], [33]. In the PCA conducted in this study, the top three PCs only explained 6.8% of the genetic variance, indicating a weak population structure among alfalfa accessions. However, the population structure cluster information clarified the genetic segregation of accessions originating from different geographical regions. For example, the accessions from Asia and South America were separated into different genetic clusters (Figure 4B), which is consistent with the results of an earlier study on the alfalfa core germplasm population [27]. These genetic clusters may be explained by the differences in the domestication and adaptations to environmental conditions among the alfalfa accessions from diverse regions. However, outcrossing and the applied breeding strategy resulted in substantial gene flow between accessions. The genes mediating adaptations to different regions may be exchanged, thereby enabling alfalfa to spread worldwide. Furthermore, the European accessions were genetically diverse, which may be related to the fact that alfalfa originated in the Caucasus region (*i.e.*, northeastern Turkey, Turkmenistan, and northwestern Iran). This phenomenon was reported previously [6], [34], [35].

Because of the weak population structure, it is easier to control false positive SNPs in alfalfa than in self-pollinated crops, including rice [36] and soybean [37]; this was reflected in our QQ plot results (Figure 5, Figure S8). Thus, alfalfa research does not require the construction of an artificial population to control the population structure. However, heterozygosity is a major problem for an alfalfa GWAS. We detected SNP markers using single-allele chromosomes. Homozygous genotypes among individuals were detected for 57% of the markers. Heterozygous genotypes make it difficult to accurately estimate the MAF and filter MAF markers. Furthermore, the GWAS method depends on a homozygous genotype for SNPs among individuals. Too many heterozygotes may lead to inaccurate GWAS results for the markers [6], [24], [38]. Although there are some challenges to conduct an alfalfa GWAS, we still identified 101 SNPs significantly associated with 27 phenotypes. The subsequent analysis of one SNP (Chr6_2__67485202) revealed differences in the allele frequency between cultivars/landraces and wild accessions. The allele frequency also differed between cultivars from different geographical regions. These findings were consistent with the domestication and crop improvement patterns of maize [39] and soybean [33]. Furthermore, the expression level of a candidate gene (Msa0924940) located close to the SNP Chr6_2__67485202 was higher in high-dormancy accessions than in low-dormancy accessions. The Msa0924940 homologs in *M. truncatula* encode NADH dehydrogenases. Some members of this gene family were reported to mediate cold and some other environment stresses in *Arabidopsis* and potato [40], [41]. Dormancy is highly correlated with low-temperature stress responses in alfalfa [42]. Accordingly, the NADH dehydrogenase encoded by Msa0924940 may be crucial for regulating the dormancy and low-temperature stress responses of alfalfa. Our gene expression analysis indicated that the expression of Msa0544870, which is located close to the salt stress-associated SNP Chr4_1__33943910 and encodes the MYM-type zinc finger protein, was induced by salt stress. However, the expression of this gene did not differ significantly between salt-tolerant and salt-sensitive accessions. The expression of a homologous zinc finger protein-coding genes in *Arachis hypogaea* was reportedly induced by salt stress, leading to enhanced salt stress tolerance [43]. Thus, Msa0544870 likely positively regulates salt stress responses in alfalfa. However, the functions of the associated genes in alfalfa need to be validated. Considered together, the SNPs identified in this study may be useful for future molecular characterization of alfalfa as well as for molecular breeding and investigations of candidate genes.

## Materials and methods

### Plant materials and genomic DNA sequencing

Fresh leaves, stems, and flowers were collected from one 5-year-old Zhongmu-4 alfalfa cultivar plant grown at the planting base, Langfang, Hebei Province, China (39.59 °N, 116.59 °E). Genomic DNA was extracted from the leaves using the PlantZol Kit (Catalog No. EE141-01, TransGen Biotech, Beijing, China). A portion of the DNA was used to construct 20-kb CLR libraries, which were sequenced using the PacBio Sequel platform (Pacific Biosciences, San Francisco, CA). Nineteen single-molecule real-time cells were sequenced. The remaining DNA was used to construct libraries for the paired-end sequencing using the Illumina NovaSeq platform (Illumina, San Diego, CA). The sequencing was completed by Biomarker Technologies Corporation (Beijing, China) and Novogene Corporation (Beijing, China).

A total of 220 alfalfa accessions, including 55 cultivars, 26 cultivated materials, 95 landraces, 4 breeding materials, 16 wild materials, and 24 materials with an unclear improvement status (Table S10), were used for a GWAS. Twenty-six of the accessions were obtained from the Medium Term Library of the National Grass Seed Resources of China, whereas 194 accessions were obtained from the U.S. National Plant Germplasm System (https://npgsweb.ars-grin.gov/gringlobal/search). The accessions were mainly from China, USA, Turkey, Afghanistan, Uzbekistan, Spain, Russia, Morocco, France, and Argentina. In 2018, the 220 accessions were grown in a field plot at the Chinese Academy of Agricultural Sciences in Langfang, Hebei Province, China according to a randomized complete block design with three replications. For each accession, five plants (*i.e.*, replicates) were grown in a single row, with 30 cm between plants and 65 cm between rows. To maintain uniform plants, they were clipped to a height of 5 cm after transplanting. All plants were clipped again at the early flowering stage (*i.e.*, when 10% of the plants started to flower). A total of 93 traits were analyzed in the GWAS, including 4 agronomic traits (fall growth, dormancy, leaf length, and leaf width), 31 nutrient-related traits, and 58 traits from the U.S. National Plant Germplasm System (31 growth-related phenotypes and 27 stress resistance-related phenotypes) (Table S11).

### Hi-C library construction and sequencing

The Hi-C libraries were constructed as previously described [44]. Young leaves collected from Zhongmu-4 plant were fixed in formaldehyde. The denatured chromatin was isolated from the fixed leaves and digested with *Hind* III. The ends of the digested fragments were labeled with biotin and ligated to form chimeric junctions. The chimeric fragments were isolated and then used to construct five Hi-C libraries that were sequenced using the Illumina NovaSeq platform to generate 150 bp paired-end reads. A total of 951 million reads (285 Gb) were produced.

### Genome estimation and assembly

The Zhongmu-4 genome size was estimated according to the *K*-mer frequency-based method using the Illumina paired-end sequencing reads by KmerGenie (v1.7051) [45]. The genome size was also determined by flow cytometry. Fresh leaves from Zhongmu-4 and Jemalong A17 (a close relative with a completely sequenced genome comprising 425 Mb) plants were finely chopped using a sharp blade in 250 μl Cystain PI Absolute P nuclei extraction buffer (Sysmex, Kobe, Japan). After incubating for 60 s, the homogenate was filtered through a strainer (50 μm pores). The filtrate was mixed with 500 μl staining buffer (50 μg/ml propidium iodide and 5 U/ml RNase A), and then incubated for 15 min at room temperature in darkness. The fluorescence intensities of the nuclear DNA were measured using the LSRFortessa platform (BD Biosciences, Franklin Lakes, NJ). The Zhongmu-4 genome size was determined by comparing the average propidium iodide adsorbing value with that of Jemalong A17. The genome size was also estimated using the Plant DNA C-values Database (https://cvalues.science.kew.org/).

The genome was assembled as follows: 1) PacBio CLR subreads were assembled into contigs using the default parameters of Canu (v1.8) [46]. The genome size was set as 3000 Mb. 2) The obtained contigs were polished using the Illumina genome data with Pilon (v1.23), interrupted by Juicer (v1.5) according to the Hi-C data, and then preliminarily assembled into scaffolds using 3D-DNA (v180419) [47], [48]. 3) The obtained scaffolds were assembled into chromosomes using the chromosome-scale autopolyploid genome assembly program ALLHiC as described on GitHub [25], [26] (https://github.com/tangerzhang/ALLHiC). The gene annotation data for *M*. *truncatula* Mt4.0v1 in the Phytozome database (v12) were used for assembling chromosomes and analyzing collinearity [22]. The GMAP program (v2013-10-28) was used to generate an allelic contig table to remove background Hi-C signals [49]. Synteny plots were drawn using MCScanX [50].

### Gene annotation

RNA-seq data and the amino acid sequences of proteins from *M*. *truncatula* (Mt4.0v1) were used for the *ab initio* prediction of genes. The RNA extracted from the leaves, stems, and flowers was reversely transcribed to cDNA, which was then used to construct paired-end libraries that were sequenced using the Illumina NovaSeq platform. The RNA-seq data were assembled using the default parameters of Trinity (v2.9.0) for the *de novo* and genome-guided assembly pipelines [51]. Two rounds of gene annotations were performed. More specifically, the assembled transcripts and homologous protein sequences were first annotated using BRAKER (v2.15), which enabled the fully automated training of the gene prediction tools GeneMark-EX (v4.61) and AUGUSTUS (v3.3.3) to analyze the RNA-seq data and/or the homologous protein sequences [52], [53], [54]. In the second round, the previously trained GeneMark and AUGUSTUS files, assembled transcripts, and homologous proteins were imported into the MAKER (v2.31.10) pipeline (http://www.yandell-lab.org/software/maker.html) to integrate the transcript evidence and the protein-coding evidence. The BUSCO program (v4) was used for evaluating annotation completeness [55]. The allelic genes were identified by AlleleFinder (https://github.com/sc-zhang/AlleleFinder).

### Repetitive sequence prediction

A *de novo* TE sequence library was constructed for the assembled genome using RepeatModeler (v2.0.1, http://www.repeatmasker.org/RepeatModeler/), which employs three *de novo* repetitive sequence annotation programs (RECON, v1.08; RepeatScout, v1.0.6; LTR_retriever, v2.8.7) to identify repetitive elements in the genome sequence. The repetitive sequence library customized by RepeatModeler was then imported into RepeatMasker (v4.0.9, http://www.repeatmasker.org/) to identify and cluster the repetitive elements. Tandem Repeat Finder (v4.09) was used to detect tandem repeats [56]. To more precisely identify LTRs, the LTR_FINDER (v1.07) and LTRharvest (a module of genometools v1.5.10) programs were employed to examine the LTRs [57], [58], [59]. Additionally, LTR_retriever (v2.8.7) was used to integrate the results of LTR_FINDER and LTRharvest. It was also used to calculate the LAI to assess the quality of the assembled genome [29].

### Phylogeny and gene family evolution analyses

The sequences of the proteins encoded by genes in the first set of subgenomes (Chrx_1) of Zhongmu-4 and 14 other plant species (*Lotus japonicus*, *Pisum sativum*, *A. thaliana*, *A. hypogaea*, *Cicer arietinum*, *Glycine max*, *Glycine soja*, *Lupinus albus*, *M. truncatula*, *Phaseolus lunatus*, *Phaseolus vulgaris*, *Trifolium pratense*, *Vigna unguiculata*, and *P. trichocarpa*) were used to analyze phylogenetic relationships using OrthoFinder (v2.3.11) [60]. On the basis of the specie phylogenetic tree and the divergence time of *A. thaliana* and *M. sativa* (106 MYA) (http://timetree.org/), an ultrametric tree (timetree) was produced using the r8s program (v1.81). The changes in gene family size in these species were analyzed using CAFE (v4.2.1) [61]. The key enzymes in the nitrogen metabolism pathway were identified by searching the KEGG database [62]. A phylogenetic tree for the GiSs encoded by genes in the first subgenomes of Zhongmu-4 and seven other species was constructed according to the Neighbor-joining method of MEGA X [63]. The genome annotation data for *L. japonicus* were downloaded from an online database (http://www.kazusa.or.jp/lotus/). The genome annotation data of *P. sativum* were downloaded from an online database (https://urgi.versailles.inra.fr/Species/Pisum). The genome annotation data for *A. thaliana*, *A. hypogaea*, *C. arietinum*, *G. max*, *G. soja*, *L. albus*, *M. truncatula*, *P. lunatus*, *P. vulgaris*, *T. pratense*, *V. unguiculata*, and *P. trichocarpa* were downloaded from the Phytozome database (https://phytozome-next.jgi.doe.gov/).

### Synteny and whole-genome duplication analyses

The syntenic blocks between the Zhongmu-4 subgenomes, as well as between the first Zhongmu-4 subgenome and the *M. truncatula* genome, were identified using MCScanX [50]. The Ka and Ks values of the syntenic paralogs were calculated using the Perl script “add_ka_and_ks_to_collinearity.pl” of MCScanX. Whole-genome alignment was performed by MUMmer4 [64].

### Gene functional annotation and classification

The protein-coding genes identified in Zhongmu-4 were functionally annotated using eggNOG-mapper (v5.0) [65] and InterProScan (v5.36-75.0) [66]. The KEGG pathways associated with the annotated genes were identified using the KEGG automatic annotation server [67].

### Resequencing and SNP calling

For each accession, young leaves (2 weeks after regrowth) were collected from a representative plant for resequencing. Total DNA was extracted from the leaves using the CWBIO Plant Genomic DNA Kit (Catalog No. CW0531M, CoWin Biosciences, Beijing, China). At least 6 μg genomic DNA from each accession was used to construct a sequencing library according to the manufacturer’s instructions (Illumina, San Diego, CA). Paired-end sequencing libraries were sequenced using the Illumina NovaSeq 6000 platform (Illumina, San Diego, CA) by Berry Genomics to generate 150-bp paired-end reads. Approximately 10 Gb sequencing data, with an average Q30 of 85%, were produced for each accession. The paired-end sequencing reads were mapped to the assembled Zhongmu-4 genome using the default parameters of BWA-MEM [68]. SAMtools was used to convert the SAM file to a BAM file and for sorting the BAM file according to the default parameters [69]. Picard was used to mark duplicate reads, whereas GenomeAnalysisTK was used to correct indels, which can be mistaken for SNPs. Additionally, SAMtools mpileup and VarScan were used to detect SNPs [70]. Furthermore, the following criteria were used to filter the SNP data using Vcftools [71]: missing rate ≤ 10%, MAF ≥ 0.05, and mean read depth ≥ 5.

### Population structure and LD analyses

The population structure was analyzed using the default parameters of Admixture [72]. The best *K* value was determined according to the CV error. A PCA was conducted using GAPIT3 [73]. The PCA results were combined with the information regarding the geographic origins of the accessions using the R package ggplot2 [74]. The LD was calculated using the default parameters of PopLDdecay [75]. The VCF file containing information regarding all 111,075 SNP markers was imported into PopLDdecay. The LD results for all accessions were used to estimate the LD in the alfalfa genome. The distance against the mean *r*^2^ within 50 kb of the LD was visualized using the R package ggplot2.

### Association mapping

A GWAS was conducted using the Blink function of GAPIT3 [73], [76]. The first three PC values were used as fixed effects in the model to correct false positives. The threshold used for detecting significant SNPs was the Bonferroni multiple test threshold (*P* = 9.00 × 10^−7^). The Blink method uses iterations to select a set of markers associated with a trait. These associated markers are fitted as covariates for testing the other markers. This method is better for controlling false positives than the kinship approach. The real and simulated data indicated that the statistical power of the Blink method is greater than that of other methods (*e.g.*, MLM and FarmCPU) [76]. Manhattan plots and QQ plots of the GWAS results were produced using the R package qqman [77].

### Gene expression verification by qRT-PCR

Genes were selected from the upstream and downstream candidate regions of the LD decay distance of significant SNPs. Alfalfa accessions with the five highest or lowest values for the evaluation of salt tolerance, fall growth, and frost damage were selected for analyzing gene expression. Seeds were imbibed at 4 °C for 2 days and then incubated under long-day conditions [16-h light (24 °C)/8-h dark (20 °C)] for 4 days. Then, the seedlings were transferred to half-strength Hoagland solution and grown for 10 days. To simulate salt stress, 14-day-old seedlings were treated with 150 mM NaCl under long-day conditions. The shoots were collected 3.5 days (84 h) later. To analyze fall dormancy-related gene expression, leaves were collected from five high-dormancy accessions and five low-dormancy accessions in October. Total RNA was extracted from the collected samples using the Mini BEST Plant RNA Extraction Kit (Catalog No. 9769, Takara, Dalian, China), and then cDNA was synthesized using the One-Step PrimeScript RT-PCR Kit (Catalog No. RR014A, Takara). The qRT-PCR analysis was performed using the TB Green Premix Ex Taq II Kit (Catalog No. RR820Q, Takara) and the ABI 7300 system (Applied Biosystems, Foster City, CA). Gene expression levels were calculated according to the mean threshold cycle (Ct) values of three biological replicates. The alfalfa *β-actin* gene was used as the internal reference control.

## Data availability

The genome assembly and gene annotation data are available online (https://figshare.com/s/fb4ba8e0b871007a9e6c). The whole-genome assembly data have also been deposited in the Genome Warehouse [78] at the National Genomics Data Center, Beijing Institute of Genomics, Chinese Academy of Sciences/ China National Center for Bioinformation (GWH: GWHBECI00000000), and are publicly accessible at https://ngdc.cncb.ac.cn/gwh/. The genome and transcriptome sequencing raw data and the population resequencing data have been deposited in the Genome Sequence Archive [79] at the National Genomics Data Center, Beijing Institute of Genomics, Chinese Academy of Sciences / China National Center for Bioinformation (GSA: CRA005190 and CRA003659, repectively), and are publicly accessible at https://ngdc.cncb.ac.cn/gsa. The genome and transcriptome sequencing raw data have also been deposited in National Center for Biotechnology Information (Project: PRJNA685277), and are publicly accessible at https://www.ncbi.nlm.nih.gov/.

## CRediT author statement

**Ruicai Long:** Conceptualization, Methodology, Software, Validation, Formal analysis, Investigation, Data curation, Writing - original draft, Visualization, Project administration, Funding acquisition. **Fan Zhang:** Methodology, Software, Validation, Formal analysis, Investigation, Data curation, Writing - original draft, Visualization. **Zhiwu Zhang:** Methodology, Validation, Writing - review & editing. **Mingna Li:** Validation, Writing - original draft. **Lin Chen:** Methodology, Visualization. **Xue Wang:** Investigation, Visualization. **Wenwen Liu:** Investigation. **Tiejun Zhang:** Investigation. **Long-Xi Yu:** Writing - review & editing. **Fei He:** Investigation. **Xueqian Jiang:** Investigation. **Xijiang Yang:** Investigation. **Changfu Yang:** Investigation. **Zhen Wang:** Methodology, Writing - review & editing, Visualization, Project administration. **Junmei Kang:** Project administration, Funding acquisition. **Qingchuan Yang:** Conceptualization, Resources, Supervision, Funding acquisition. All authors have read and approved the final manuscript.

## Competing interests

The authors have declared no competing interests.

## Supplementary material

Supplementary Table S1 **PI-A values analyzed by flow cytometry and estimated genome size**

Supplementary Table S2 **Sequencing data information**

Supplementary Table S3 **Contigs assembled using Canu and contigs corrected on the basis of Hi-C reads**

Supplementary Table S4 **Summary of the assembled Zhongmu-4 genome**

Supplementary Table S5 **BUSCO analysis for the assembled Zhongmu-4 genome and the annotated proteins**

Supplementary Table S6 **Summary of the gene annotation results**

Supplementary Table S7 **Gene copy numbers of some enzymes involved in the nitrogen metabolism pathway in the first subgenome of Zhongmu-4 and seven other species**

Supplementary Table S8 **Information regarding the SNPs associated with 27 traits**

Supplementary Table S9 **Comparison of the basic assembly parameters of the Zhongmu-4 genome and two other alfalfa genomes**

Supplementary Table S10 **Basic information regarding 220 alfalfa accessions**

Supplementary Table S11 **Phenotypic information regarding 93 agronomic traits**
