# Supplementary material for: Genome Assembly of Alfalfa Cultivar Zhongmu-4 and Identification of SNPs Associated with Agronomic Traits
Source: Genomics Proteomics Bioinformatics. 2022 Jan 13;20(1):14–28. doi: 10.1016/j.gpb.2022.01.002 (PMC9510860; doi:10.1016/j.gpb.2022.01.002)
Supplement: Supplementary Table S1 — PI-A values analyzed by flow cytometry and estimated genome size [file mmc1.docx]

**Table S1 PI-A values analyzed by flow cytometry and estimated genome size**

| **Repeat** | **PI-A (Msa)** | **PI-A (Mtr)** | **FC of PI-A (Msa/Mtr)** | **Average FC of PI-A** | **Monoploid genome size of Mtr (Mb)** | **Estimated** **tetraploid** **genome size of Msa (Mb)** |
| --- | --- | --- | --- | --- | --- | --- |
| Repeat 1 | 177,794 | 49,029 | 3.63 | 3.61 | 390–425 | 2814–3068 |
| Repeat 2 | 189,027 | 52,731 | 3.58 |  |  |  |
| Repeat 3 | 182,224 | 50,426 | 3.61 |  |  |  |

*Note*: Msa, *M. sativa* cultivar Zhongmu-4; Mtr, *M. truncatula* ecotype Jemalong A17; FC, fold change; PI-A, propidium iodide adsorbing.
