# Supplementary material for: Genome Assembly of Alfalfa Cultivar Zhongmu-4 and Identification of SNPs Associated with Agronomic Traits
Source: Genomics Proteomics Bioinformatics. 2022 Jan 13;20(1):14–28. doi: 10.1016/j.gpb.2022.01.002 (PMC9510860; doi:10.1016/j.gpb.2022.01.002)
Supplement: Supplementary Table S2 — Sequencing data information [file mmc2.docx]

**Table S2 Sequencing data information**

| **Data type** | **Data size (Gb)** | **Depth (×)** | **N_50_ (bp)** |
| --- | --- | --- | --- |
| PacBio CLR | 262 | 85 | 29,708 |
| Illumina read (genome) | 168 | 54 | 150 |
| Illumina read (Hi-C) | 285 | 92 | 150 |
| Illumina read (transcriptome) | 26 | - | 150 |

*Note*: The estimated whole tetraploid genome size of Zhongmu-4 was set as 3.1 Gb. CLR, continuous long reads; Hi-C, High-through chromosome conformation capture.
