# Supplementary material for: Genome Assembly of Alfalfa Cultivar Zhongmu-4 and Identification of SNPs Associated with Agronomic Traits
Source: Genomics Proteomics Bioinformatics. 2022 Jan 13;20(1):14–28. doi: 10.1016/j.gpb.2022.01.002 (PMC9510860; doi:10.1016/j.gpb.2022.01.002)
Supplement: Supplementary Table S3 — Contigs assembled using Canu and contigs corrected on the basis of Hi-C reads [file mmc3.docx]

**Table S3** **Contigs assembled using Canu and contigs corrected on the basis of Hi-C** **reads**

| **Contig** | **Number of contig** | **Total Length (Gb)** | **N50 (kb)** | **GC (%)** |
| --- | --- | --- | --- | --- |
| Canu assembled contigs | 5000 | 2.74 | 2060 | 34.20 |
| Hi-C corrected contigs | 49,967 | 2.74 | 94 | 34.20 |
