# Supplementary material for: Genome Assembly of Alfalfa Cultivar Zhongmu-4 and Identification of SNPs Associated with Agronomic Traits
Source: Genomics Proteomics Bioinformatics. 2022 Jan 13;20(1):14–28. doi: 10.1016/j.gpb.2022.01.002 (PMC9510860; doi:10.1016/j.gpb.2022.01.002)
Supplement: Supplementary Table S4 — Summary of the assembled Zhongmu-4 genome [file mmc4.docx]

**Table S4 Summary of the assembled Zhongmu-4 genome**

| **Category** | **Contig number** | **Length (bp)** |
| --- | --- | --- |
| Chr1_1 | 1405 | 81,475,151 |
| Chr1_2 | 1466 | 79,614,812 |
| Chr1_3 | 1358 | 77,152,361 |
| Chr1_4 | 1439 | 76,635,544 |
| Chr2_1 | 1335 | 82,498,830 |
| Chr2_2 | 1141 | 70,773,673 |
| Chr2_3 | 1139 | 66,739,748 |
| Chr2_4 | 1190 | 65,143,524 |
| Chr3_1 | 1678 | 94,727,156 |
| Chr3_2 | 1662 | 91,622,233 |
| Chr3_3 | 1796 | 91,676,550 |
| Chr3_4 | 1483 | 82,148,590 |
| Chr4_1 | 1537 | 87,806,598 |
| Chr4_2 | 1277 | 76,139,727 |
| Chr4_3 | 1321 | 74,558,508 |
| Chr4_4 | 1455 | 82,903,785 |
| Chr5_1 | 1052 | 71,963,113 |
| Chr5_2 | 1529 | 78,992,507 |
| Chr5_3 | 1166 | 69,949,269 |
| Chr5_4 | 1275 | 70,080,956 |
| Chr6_1 | 1938 | 104,280,083 |
| Chr6_2 | 1538 | 83,446,217 |
| Chr6_3 | 1875 | 99,942,479 |
| Chr6_4 | 1789 | 91,272,922 |
| Chr7_1 | 1174 | 81,116,394 |
| Chr7_2 | 1528 | 88,039,813 |
| Chr7_3 | 1577 | 87,612,856 |
| Chr7_4 | 1110 | 64,180,167 |
| Chr8_1 | 1311 | 91,953,106 |
| Chr8_2 | 1186 | 73,305,697 |
| Chr8_3 | 1252 | 69,667,704 |
| Chr8_4 | 1267 | 56,218,238 |
| unanchored contigs | 4864 | 179,289,811 |
| total contigs | 49,967 | 2,742,928,122 |
| anchor rate (%) | 93.45 | |
