# Supplementary material for: Genome Assembly of Alfalfa Cultivar Zhongmu-4 and Identification of SNPs Associated with Agronomic Traits
Source: Genomics Proteomics Bioinformatics. 2022 Jan 13;20(1):14–28. doi: 10.1016/j.gpb.2022.01.002 (PMC9510860; doi:10.1016/j.gpb.2022.01.002)
Supplement: Supplementary Table S5 — BUSCO analysis for the assembled Zhongmu-4 genome and the annotated proteins [file mmc5.docx]

**Table S5 BUSCO analysis for the assembled Zhongmu-4 genome and the annotated proteins**

| **Search mode** | **Complete BUSCOs** | **Single-copy BUSCOs** | **Duplicated BUSCOs** | **Fragmented BUSCOs** | **Missing BUSCOs** | **Total searched BUSCOs** |
| --- | --- | --- | --- | --- | --- | --- |
| Assembled genome | 1588(98.4%) | 120(7.4%) | 1468(91.0%) | 5(0.3%) | 21(1.3%) | 1614 |
| Annotated proteins | 1552(96.1%) | 99(6.1%) | 1453(90.0%) | 17(1.1%) | 45(2.8%) | 1614 |

*Note*: BUSCO, benchmarking universal single-copy orthologs.
