# Supplementary material for: Genome Assembly of Alfalfa Cultivar Zhongmu-4 and Identification of SNPs Associated with Agronomic Traits
Source: Genomics Proteomics Bioinformatics. 2022 Jan 13;20(1):14–28. doi: 10.1016/j.gpb.2022.01.002 (PMC9510860; doi:10.1016/j.gpb.2022.01.002)
Supplement: Supplementary Table S6 — Summary of the gene annotation results [file mmc6.docx]

**Table S****6** **Summary of the gene annotation results**

| **Category** | **Total length** | **Annotated gene number** | **CDS length** | **CDS length percent** | **cDNA length** | **cDNA length percent** | **gene length** | **gene length percent** |
| --- | --- | --- | --- | --- | --- | --- | --- | --- |
| Chr1_1 | 81,475,151 | 4808 | 5,693,277 | 6.99% | 7,129,491 | 8.75% | 19,561,034 | 24.01% |
| Chr1_2 | 79,614,812 | 4729 | 5,737,290 | 7.21% | 7,166,829 | 9.00% | 19,579,851 | 24.59% |
| Chr1_3 | 77,152,361 | 4626 | 5,409,660 | 7.01% | 6,745,112 | 8.74% | 18,527,290 | 24.01% |
| Chr1_4 | 76,635,544 | 4415 | 5,305,050 | 6.92% | 6,621,100 | 8.64% | 18,348,694 | 23.94% |
| Chr2_1 | 82,498,830 | 4288 | 4,963,065 | 6.02% | 6,138,066 | 7.44% | 17,188,537 | 20.83% |
| Chr2_2 | 70,773,673 | 4060 | 4,685,250 | 6.62% | 5,761,616 | 8.14% | 16,059,709 | 22.69% |
| Chr2_3 | 66,739,748 | 3787 | 4,434,615 | 6.64% | 5,500,904 | 8.24% | 15,482,882 | 23.20% |
| Chr2_4 | 6,5143,524 | 3531 | 4,128,648 | 6.34% | 5,167,416 | 7.93% | 14,746,191 | 22.64% |
| Chr3_1 | 94,727,156 | 4727 | 5,614,509 | 5.93% | 6,959,131 | 7.35% | 19,189,192 | 20.26% |
| Chr3_2 | 91,622,233 | 4766 | 5,684,358 | 6.20% | 7,058,504 | 7.70% | 19,411,778 | 21.19% |
| Chr3_3 | 91,676,550 | 4598 | 5,524,530 | 6.03% | 6,833,366 | 7.45% | 18,922,820 | 20.64% |
| Chr3_4 | 82,148,590 | 4427 | 5,283,189 | 6.43% | 6,502,009 | 7.91% | 17,798,884 | 21.67% |
| Chr4_1 | 87,806,598 | 5446 | 6,500,250 | 7.40% | 8,263,854 | 9.41% | 22,625,002 | 25.77% |
| Chr4_2 | 76,139,727 | 4089 | 4,796,601 | 6.30% | 6,021,828 | 7.91% | 16,238,061 | 21.33% |
| Chr4_3 | 74,558,508 | 3938 | 4,639,266 | 6.22% | 5,839,379 | 7.83% | 16,303,257 | 21.87% |
| Chr4_4 | 82,903,785 | 4668 | 5,571,273 | 6.72% | 6,993,784 | 8.44% | 18,989,225 | 22.91% |
| Chr5_1 | 71,963,113 | 3847 | 4,238,202 | 5.89% | 5,304,540 | 7.37% | 14,792,582 | 20.56% |
| Chr5_2 | 78,992,507 | 4212 | 4,750,587 | 6.01% | 5,917,249 | 7.49% | 16,482,853 | 20.87% |
| Chr5_3 | 69,949,269 | 3836 | 4,403,655 | 6.30% | 5,494,350 | 7.85% | 15,393,684 | 22.01% |
| Chr5_4 | 70,080,956 | 3930 | 4,416,939 | 6.30% | 5,545,328 | 7.91% | 15,349,274 | 21.90% |
| Chr6_1 | 104,280,083 | 3563 | 3,805,755 | 3.65% | 4,581,904 | 4.39% | 13,354,937 | 12.81% |
| Chr6_2 | 83,446,217 | 2906 | 3,050,577 | 3.66% | 3,662,576 | 4.39% | 10,773,400 | 12.91% |
| Chr6_3 | 99,942,479 | 3669 | 3,903,099 | 3.91% | 4,706,576 | 4.71% | 14,558,121 | 14.57% |
| Chr6_4 | 91,272,922 | 3437 | 3,602,808 | 3.95% | 4,331,539 | 4.75% | 13,033,427 | 14.28% |
| Chr7_1 | 81,116,394 | 3978 | 4,636,509 | 5.72% | 5,735,609 | 7.07% | 15,987,591 | 19.71% |
| Chr7_2 | 88,039,813 | 4448 | 5,106,495 | 5.80% | 6,301,766 | 7.16% | 17,297,382 | 19.65% |
| Chr7_3 | 87,612,856 | 4649 | 5,330,322 | 6.08% | 6,655,169 | 7.60% | 18,612,750 | 21.24% |
| Chr7_4 | 64,180,167 | 3698 | 4,350,816 | 6.78% | 5,380,309 | 8.38% | 14,669,171 | 22.86% |
| Chr8_1 | 91,953,106 | 5540 | 6,691,755 | 7.28% | 8,403,948 | 9.14% | 23,365,355 | 25.41% |
| Chr8_2 | 73,305,697 | 4167 | 4,973,262 | 6.78% | 6,212,684 | 8.48% | 17,503,687 | 23.88% |
| Chr8_3 | 69,667,704 | 3802 | 4,475,643 | 6.42% | 5,529,124 | 7.94% | 15,744,350 | 22.60% |
| Chr8_4 | 56,218,238 | 2911 | 3,592,509 | 6.39% | 4,464,161 | 7.94% | 12,604,053 | 22.42% |
| unanchored contigs | 179,289,811 | 13,208 | 14,623,239 | 8.16% | 18,102,100 | 10.10% | 45,185,928 | 25.20% |
| total | 2,742,928,122 | 146,704 | 169,923,003 | 6.19% | 211,031,321 | 7.69% | 583,680,952 | 21.28% |

*Note*: CDS, coding sequence.
