# Supplementary material for: Genome Assembly of Alfalfa Cultivar Zhongmu-4 and Identification of SNPs Associated with Agronomic Traits
Source: Genomics Proteomics Bioinformatics. 2022 Jan 13;20(1):14–28. doi: 10.1016/j.gpb.2022.01.002 (PMC9510860; doi:10.1016/j.gpb.2022.01.002)
Supplement: Supplementary Table S7 — Gene copy numbers of some enzymes involved in the nitrogen metabolism pathway in the first subgenome of Zhongmu-4 and seven other species [file mmc7.docx]

**Table S7 Gene copy numbers of some enzymes involved in the nitrogen metabolism pathway in the first subgenome of Zhongmu-4 and seven other species**

| **Species** | **Nrt** | **NR** | **NIR** | **GDH** | **GiS** | **GaS** |
| --- | --- | --- | --- | --- | --- | --- |
| *M. sativa* | 3 | 3 | 2 | 10 | 8 | 3 |
| *M. truncatula* | 3 | 3 | 1 | 5 | 5 | 2 |
| *G. max* | 5 | 4 | 2 | 9 | 8 | 3 |
| *A. thaliana* | 7 | 2 | 1 | 4 | 6 | 3 |
| *Z. mays* | 5 | 5 | 2 | 3 | 6 | 3 |
| *O. sativa* | 4 | 3 | 2 | 4 | 4 | 3 |
| *V.* *vinifera* | 5 | 1 | 1 | 5 | 5 | 2 |
| *P. trichocarpa* | 6 | 2 | 1 | 5 | 8 | 3 |

*Note*: Nrt, nitrate transporter; NR, nitrate reductase; NIR, nitrite reductase; GDH, glutamate dehydrogenase; GiS, glutamine synthetase; GaS, glutamate synthetase.
