# Supplementary material for: Genome Assembly of Alfalfa Cultivar Zhongmu-4 and Identification of SNPs Associated with Agronomic Traits
Source: Genomics Proteomics Bioinformatics. 2022 Jan 13;20(1):14–28. doi: 10.1016/j.gpb.2022.01.002 (PMC9510860; doi:10.1016/j.gpb.2022.01.002)
Supplement: Supplementary Table S9 — Comparison of the basic assembly parameters of the Zhongmu-4 genome and two other alfalfa genomes [file mmc9.docx]

| **Plant material** | **Sequencing platform** | **Assembled genome size** | **Assembly type** | **N_50_** | **Annotated genes** | **BUSCO** | **Anchor rate** |
| --- | --- | --- | --- | --- | --- | --- | --- |
| Zhongmu-4 | Pacbio | 2.74 Gb | Allele-aware chromosome | 2.06 Mb | 146,704 | 98.40% | 93.40% |
| Xinjiangdaye [27] | Pacbio & Nanopore | 3.15 Gb | Allele-aware chromosome | 0.46 Mb | 164,632 | 97.20% | 86.90% |
| Zhongmu-1 [28] | Pacbio | 816 Mb | Haplotype | 3.92 Mb | 49,165 | 93.30% | 97.30% |

**Table S9**  **Comparison of the basic assembly parameters of the Zhongmu-4 genome and two other alfalfa genomes**
